# Nephrotic Syndrome in a Retroviral Disease Due to AA Amyloidosis: A Rare Presentation

**DOI:** 10.7759/cureus.44928

**Published:** 2023-09-08

**Authors:** Pranjal Kashiv, Shubham Dubey, Kapil N Sejpal, Prasad Gurjar, Vrushali Mahajan, Amit Pasari, Manish Balwani

**Affiliations:** 1 Department of Nephrology, Jawaharlal Nehru Medical College, Datta Meghe Institute of Higher Education and Research, Wardha, IND; 2 Department of Pathology, Alexis Multispecialty Hospital, Nagpur, IND

**Keywords:** hiv associated nephropathy (hivan), hiv-associated nephropathy, haart, hiv, aa amyloidosis, nephrotic syndrome

## Abstract

Kidney disease poses a significant burden on individuals with HIV infection. In the pre-ART era, HIV-associated nephropathy (HIVAN) was the most common renal pathology identified in individuals with HIV. However, the widespread use of ART has led to changes in the spectrum of renal pathologies associated with HIV. HIV infection is an unclear cause of AA amyloidosis. Here, we report a rare case of an HIV-positive patient presenting with nephrotic syndrome which turned out to be AA amyloidosis on renal biopsy.

## Introduction

In 2019, it was estimated that around 38 million people were living with HIV [1]. Despite widely available highly efficient anti-retroviral therapy, HIV remains to be a chronic infection with immune activation [2]. The incidence of AA amyloidosis is estimated to be 1-2 million cases [3] and may be higher in low or low-middle-income countries due to economic constraints and scarce rheumatology services. Chronic inflammation and infections are the most common causes of AA amyloidosis. HIV is retained as a rare direct cause of AA amyloidosis and most of the available literature points towards an underlying chronic infection or IV drug abuse as the cause of AA amyloidosis in HIV. 

## Case presentation

A 40-year-old male with no previous co-morbidities was admitted to our hospital with complaints of bilateral pedal edema and reduced urine output for a duration of 14 days. On examination, he was afebrile and normotensive with a blood pressure of 110/70 mm Hg. A general examination revealed bilateral pitting pedal edema up to the level of the knee. On evaluation, he was found to have nephrotic range proteinuria, hypoalbuminemia, hypercholesterolemia, and a serum creatinine of 7.4mg/dl (Table 1). There was no history of alternative medication intake. Further evaluation showed positivity for HIV by ELISA with a CD4 count of 364 cells/cumm.

**Table 1:** Laboratory investigations

Investigation	Result
Serum creatinine	7.4 mg/dl
Haemoglobin	8 gm/dl
Total leukocyte count	7800 cells/cumm
Platelet count	3.7 lacs/cumm
Total protein	4.3gm/dl
Serum albumin	1.8 gm/dl
Total cholesterol	250 mg/dl
Triglycerides	380 mg/dl
Urine routine	3+ protein, No RBCs
Urine PC ratio	8.68

**Figure 1:**
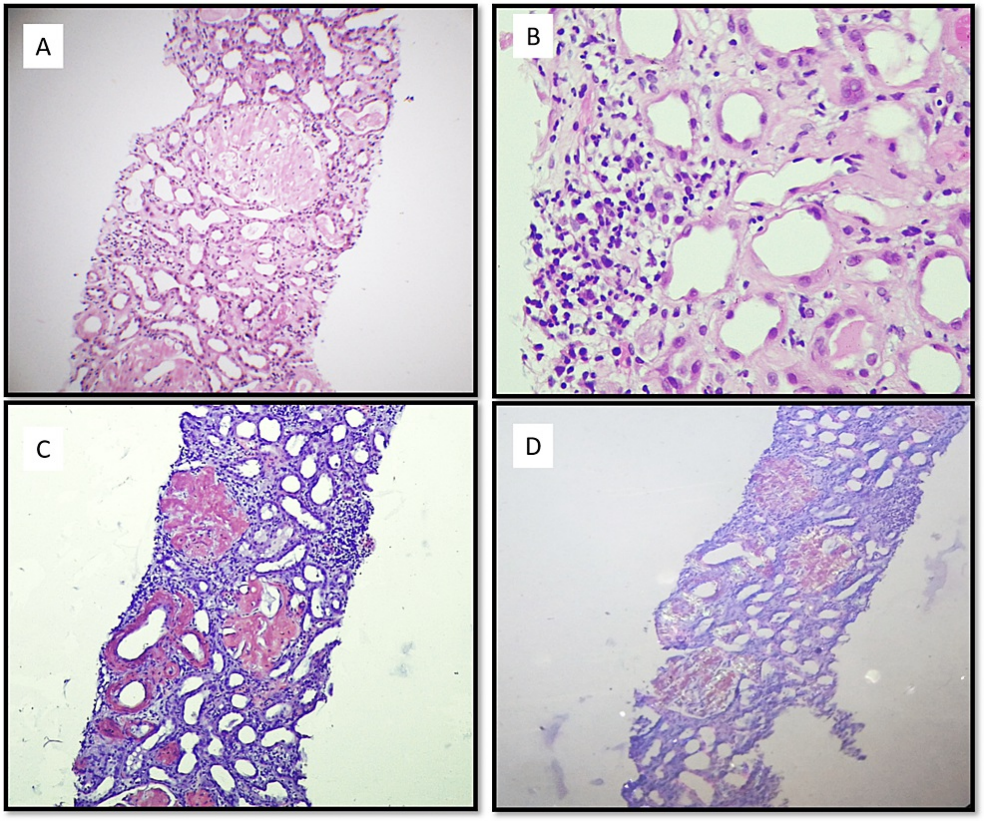
(A) H & E 10X - Microphotograph showing renal cortical tissue with glomerular deposits of amyloid. (B) H & E 40X - Microphotograph showing prominent tubular injury with simplified epithelium and lymphoplasmacytic interstitial inflammation. (C) Congo red 10X - Microphotographs showing congophilic deposits in the glomeruli, blood vessels and focally in the interstitium. (D) Congo red 10X after polarization - Microphotograph showing apple-green birefringence in the congophilic deposits.

**Figure 2:**
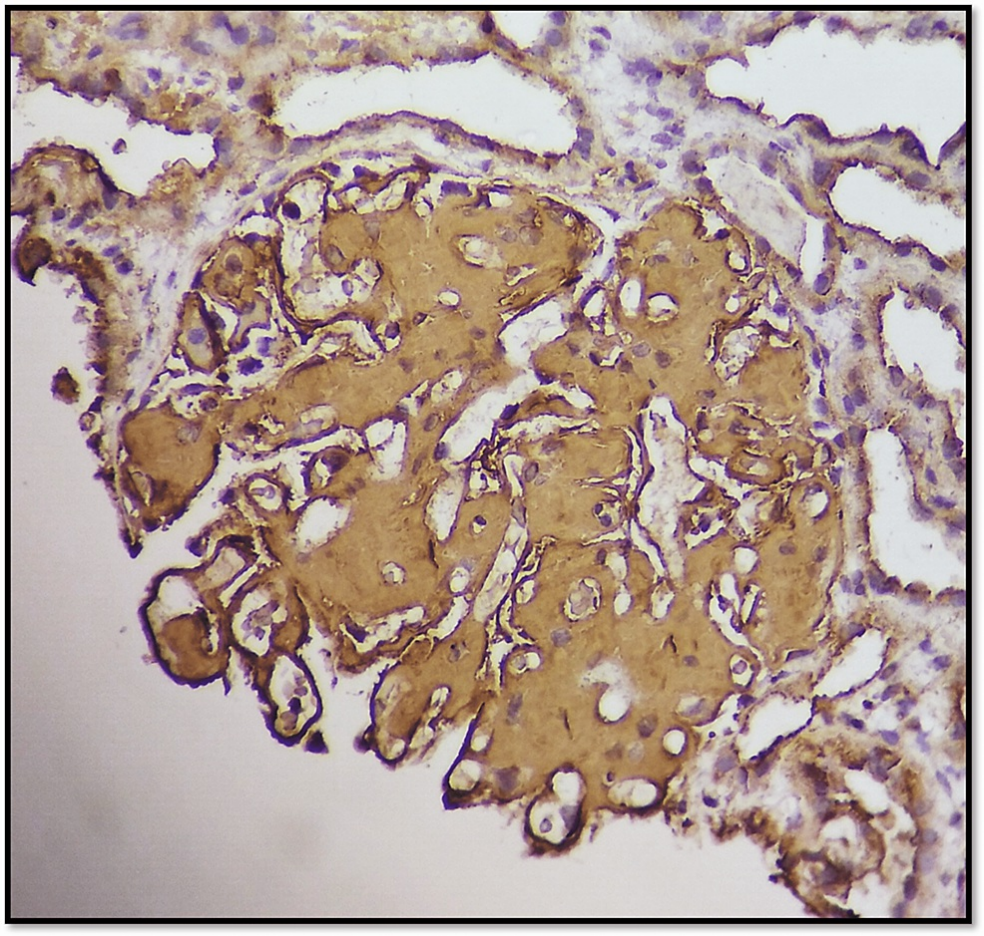
SAA IHC 40X- Microphotograph showing amyloid deposits positive for serum amyloid A protein.

He underwent two sessions of hemodialysis followed by a renal biopsy. Renal biopsy (Figures 1-2) showed 16 glomeruli, all of which appeared markedly enlarged with diffuse mesangial expansion by pale eosinophilic material and were periodic acid-Schiff stain (PAS) negative, and silver stain negative for congophilic material. There was variable staining on trichrome stain and showed apple green birefringence on polarization on a Congo red-stained section confirming it to be amyloid. There was marked acute tubular necrosis with dense interstitial inflammatory infiltrate composed of lymphocytes, plasma cells, and few a eosinophils. Immunofluorescence was negative. IHC for SAA was positive, confirming it to be AA amyloidosis. 

In view of the unclear direct association of HIV with AA amyloidosis, he was screened for underlying chronic infections. His sputum for tuberculosis by cartridge-based nucleic acid amplification testing (CBNAAT) was negative and his chest X-ray was not suggestive of any infectious etiology. His blood cultures for bacterial and fungal growth were negative. The urine culture was sterile. He also underwent a non-contrast CT of the chest and abdomen which was normal. However, his upper GI endoscopy revealed oesophageal candidiasis. He was initiated on ART (dolutegravir, abacavir, and lamivudine), fluconazole, and 0.5mg/kg steroids in view of significant interstitial inflammation. The patient’s urine output gradually improved over the next few days and he was non-oliguric on discharge.

## Discussion

Although AA amyloidosis is a systemic disease, its presentation is dominated by kidney involvement (>80% of cases) [4]. The majority of patients present with nephrotic syndrome and renal dysfunction. If left untreated, it may lead to ESRD in the majority [4]. GFR <35ml/min and proteinuria >4gm/day are independently associated with inferior renal survival in AA amyloidosis. Also, renal function deteriorates less rapidly in the absence of amyloid deposition in the glomerulus [5].

In AA amyloid kidney disease, the amyloid protein accumulates in the renal parenchyma, leading to a decrease in glomerular filtration and proteinuria [6]. This occurs when the amyloid protein clumps together, damaging the tissue and direct tissue toxicity caused by amyloid precursor proteins [7]. Serum AA, an acute phase reactant, is processed by macrophages and circulates in blood on high-density lipoproteins [8]. Different variations of the serum AA gene and high-density lipoprotein composition likely explain the vast range of clinical manifestations of AA amyloidosis [9]. Higher levels of serum AA are associated with more amyloid deposits and worse kidney function, as found by Gillmore et al [10]. Patients with serum AA levels above 50mg/L had more severe end-organ dysfunction.

The exact mechanism of amyloidosis in HIV is unknown. However, reduced IL-2 levels during HIV infection may result in the generation of pro-inflammatory cytokines which might cause the production of these abnormal proteins [11].

In a recent case series of 19 cases of AA amyloidosis in the setting of HIV by Breillat et al., the clinical presentation was that of nephrotic syndrome in 94% of patients. Progression to chronic kidney disease was seen in 64.7% of the cases. Uncontrolled HIV infection with CD4 count<400/cumm was observed in 71.4% of the patients. Repeated or chronic bacterial or fungal infection was found in 47% of cases and a history of parenteral drug use in 55% of patients. In three of the 19 cases, no underlying cause could be found other than HIV infection [12].

## Conclusions

AA amyloidosis is a rare cause of nephrotic syndrome in HIV infection. It is associated with a high risk of progression to CKD and ESRD. Though screening for the underlying infection is warranted, HIV infection solely leading to AA amyloidosis is rare and seldom reported in the literature. Treatment is mainly directed towards underlying chronic infection and HIV.
